# Comparative analysis of inflight transmission of SARS-CoV-2, influenza, and SARS-CoV-1

**DOI:** 10.1017/S0950268823001012

**Published:** 2023-06-23

**Authors:** Yingjie Luo, Yuguo Li, Shenglan Xiao, Hao Lei

**Affiliations:** 1 School of Public Health (Shenzhen), Sun Yat-sen University, Guangzhou, P. R. China; 2 School of Public Health (Shenzhen), Shenzhen Campus of Sun Yat-sen University, Shenzhen, P. R. China; 3 Department of Mechanical Engineering, The University of Hong Kong, Hong Kong SAR, P. R. China; 4 School of Public Health, Zhejiang University, Hangzhou, P. R. China

**Keywords:** inflight outbreaks, influenza, risk ratio, SARS-CoV-1, SARS-CoV-2, transmission route

## Abstract

The aim of this study is to evaluate the infection risk of aircraft passengers seated within and beyond two rows of the index case(s) of the severe acute respiratory syndrome coronavirus 2 (SARS-CoV-2), influenza A(H1N1)pdm09 virus, and SARS-CoV-1. PubMed databases were searched for articles containing information on air travel–related transmission of SARS-CoV-2, influenza A(H1N1)pdm09 virus, and SARS-CoV-1 infections. We performed a meta-analysis of inflight infection data. In the eight flights where the attack rate could be calculated, the inflight SARS-CoV-2 attack rates ranged from 2.6% to 16.1%. The risk ratios of infection for passengers seated within and outside the two rows of the index cases were 5.64 (95% confidence interval (CI):1.94–16.40) in SARS-CoV-2 outbreaks, 4.26 (95% CI:1.08–16.81) in the influenza A(H1N1)pdm09 virus outbreaks, and 1.91 (95% CI:0.80–4.55) in SARS-CoV-1 outbreaks. Furthermore, we found no significant difference between the attack rates of SARS-CoV-2 in flights where the passengers were wearing masks and those where they were not (*p* = 0.22). The spatial distribution of inflight SARS-CoV-2 outbreaks was more similar to that of the influenza A(H1N1)pdm09 virus outbreaks than to that of SARS-CoV-1. Given the high proportion of asymptomatic or pre-symptomatic infection in SARS-CoV-2 transmission, we hypothesised that the proximity transmission, especially short-range airborne route, might play an important role in the inflight SARS-CoV-2 transmission.

## Introduction

Since the worldwide pandemic of the severe acute respiratory syndrome coronavirus-2 (SARS-CoV-2), as of April 26, 2023, there have been more than 764 million confirmed cases and 6.91 million deaths globally [1]. Airplane cabins can be a high-risk environment for respiratory virus transmission because of their high occupant density and relatively long exposure times [2]. Air travel has contributed to the transmission of the coronavirus disease 2019 (COVID-19) [3]. Although rarely reported and hard to accurately assess, inflight transmission of diseases has caught much attention.

Aircraft cabins have several characteristics that may facilitate the spread of infection, such as an enclosed space, finite ventilation, re-circulating air, and long exposure times. On the other hand, these characteristics make the airplane cabin an ideal place for exploring the transmission routes of infections. Due to the fixed seating arrangement in the airplane cabin, the spatial distribution of secondary cases can be clearly retraced after the outbreak. At the same time, the temporal and spatial variations of airplane cabins are more stable than other environments.

At present, the three routes of airborne, droplet, and fomite transmission are considered to be the main modes of transmission of respiratory infectious diseases [4]. Infected individuals release droplets containing pathogens from the mouth, mainly through respiratory activities such as breathing, talking, and coughing. Susceptible individuals can inhale small-diameter aerosol or droplet nuclei (<30 μm) to cause infection (airborne transmission) [5, 6]. Larger-diameter droplets (>100 μm) can splash and deposit on the facial mucosa of susceptible individuals and cause infection (droplet transmission) [7]. Additionally, susceptible individuals can contract the infection by touching contaminated surfaces and subsequently touching their facial mucous membranes (fomite transmission) [8]. However, the relative importance of the three routes, which is fundamental to developing effective preventive strategies, remains controversial.

Recently, SARS-CoV-2 has been compared with some influenza viruses in terms of pathogenesis, host response, and pandemic pattern [9]. Although SARS-CoV-2 is a type of coronavirus, its transmission speed, scope, and characteristics are similar to the influenza A(H1N1)pdm09 virus in contrast to the severe acute respiratory syndrome virus (SARS-CoV-1). To test the hypothesis that SARS-CoV-2 has a transmission route like the influenza A(H1N1)pdm09 virus, rather than SARS-CoV-1, we conducted a comparative analysis of flight outbreaks of the three viruses, calculating the infection risk for passengers seated within and beyond two rows of the index patient(s), to explore the dominant transmission route of the three viruses during flight.

## Methods

### Literature search and study selection

According to the guidelines of the Preferred Reporting Items for Systematic Reviews and Meta-Analyses (PRISMA) [10], we systematically searched the PubMed database for articles containing information on the transmission of SARS-CoV-2 infection associated with air travel between 1 January 2020 and 2 July 2022. The search strategy is described in the Supplementary Material. Two authors (H.L. and S.X.) independently screened the titles and abstracts of all the articles and reviewed the full text of relevant articles. Studies investigating the spread of SARS-CoV-2 in flights were included. Studies on aircraft carriers, reporting the same outbreaks, summarising multiple flight transmissions, or missing key information were excluded. The data of the influenza A(H1N1)pdm09 virus and SARS-CoV-1 outbreaks were obtained from systematic literature reviews [4, 11].

### Data extraction

Three authors (H.L., S.X., and Y.Luo) independently extracted relevant data from the publications and cross-checked them. Discrepancies were resolved through joint reading and comprehensive discussions of the study among the three authors. The summary table recorded the extracted data that included the first authors of publications, flight departure dates, flight durations, the numbers of passengers aboard, passengers traced, index cases, secondary cases, attack rates, and use of masks. Furthermore, we conducted an evaluation of the level of evidence in the included studies. The assessment primarily relied on the confirmation method employed for index cases and secondary cases. Studies that employed epidemiological investigations and whole-genome sequencing to confirm both index cases and secondary cases were assigned a high level of evidence, indicating the highest level of credibility. Studies with a medium level of evidence had clear index cases and relied on epidemiological investigations to confirm secondary cases. Studies that involved suspected index cases were assigned a low level of evidence. Since the distance between each row of seats in the tourist class of an airplane is about 0.8 meters, the distance between two rows of passengers is just within the exposure range of droplet transmission. Thus, this study explored secondary cases within two rows and the attack rates of passengers within two rows.

### Case definitions

The index case was defined as a passenger who presented with COVID-19 symptoms or a positive laboratory test for SARS-CoV-2 RNA within 14 days before the flight or on arrival. The secondary case was defined as a passenger who did not present with laboratory-confirmed COVID-19 before the flight but developed it within 14 days after the flight or whose specimen yielded a viral genomic sequence close to the strain of the index case(s). Case definitions of the influenza A(H1N1)pdm09 virus and SARS-CoV-1 are provided in detail in the studies by Lei et al. [4] and Browne et al. [11].

### Data analysis

We defined the passengers seated in two rows of the front and back of the index case as the exposure group and the passengers outside the two rows as the control group. The overall attack rate of all flights was calculated by dividing the total number of secondary cases by the total number of susceptible passengers or the number of traced susceptible passengers when the total number of flight passengers was unknown. The attack rate for the exposure group was calculated as the number of secondary cases within two rows divided by the total number of susceptible passengers within two rows or the number of traced susceptible passengers within two rows. Additionally, we used a two-sided chi-squared test to determine whether there was a difference in SARS-CoV-2 attack rates between flights where passengers were wearing masks and flights where they were not.

Finally, we compared the infection risk of passengers inside and outside two rows of the index case in flight outbreaks of SARS-CoV-2, influenza A (H1N1)pdm09 virus, and SARS-CoV-1 by meta-analysis. Considering the heterogeneity among the included studies of SARS-CoV-2 (*I* ^2^ > 50%), a random effects model was chosen to calculate the combined effect value.

## Results

### Literature search and study selection

**Figure 1.**
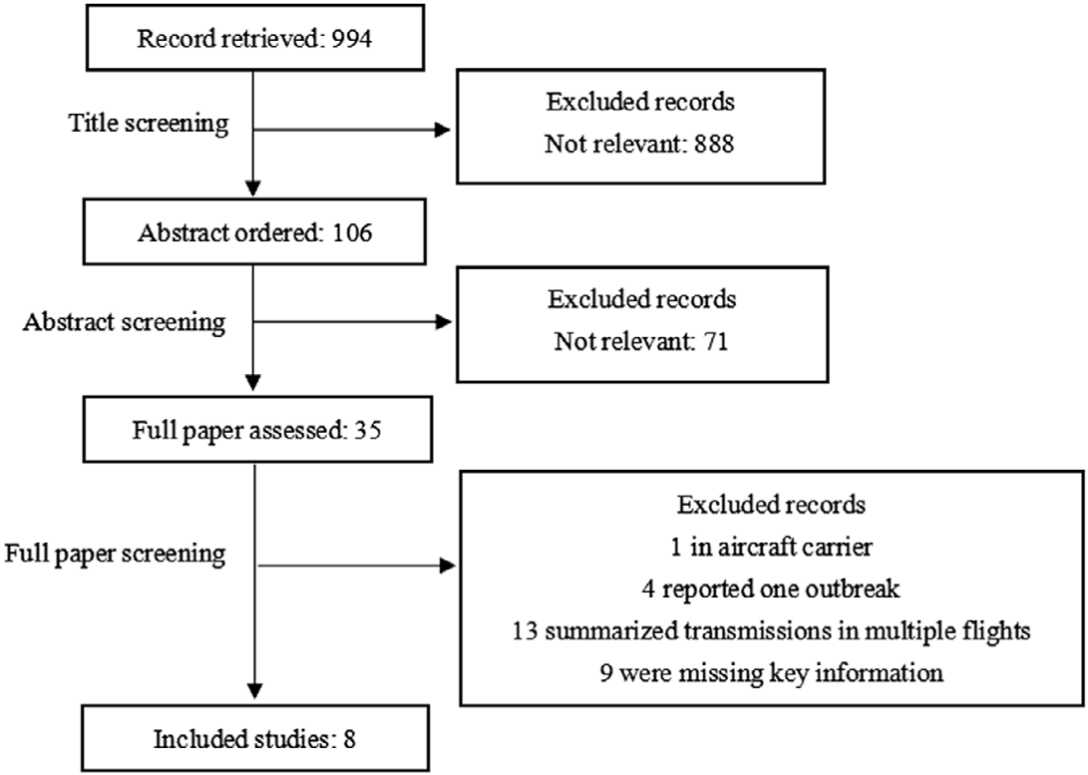
Flow diagram of the literature review of inflight SARS-CoV-2 transmission.

Figure 1 illustrates the search process. We searched for 994 related articles based on the search criteria, and 35 articles were retained after title and abstract screening. We excluded 1 article reporting the inflight SARS-CoV-2 infection of the aircraft carrier plane, 4 articles reporting the same outbreaks, 13 articles summarising transmissions in multiple flights, and 9 articles missing key information. The search results included eight inflight outbreaks [2, 12–18] occurring from 24 January 2020 to 9 June 2021. Table 1 provides detailed information about the eight flight outbreaks. In addition, the summary of the influenza A(H1N1)pdm09 virus and SARS-CoV-1 inflight outbreaks are shown in Supplementary Tables S1 and S2.

**Table 1.** Summary of inflight SARS-CoV-2 transmission from the literature

First author	Departure date	Flight duration	Passengers aboard	Passengers traced	Index cases	Secondary cases identified	Attack rate (%)	Secondary cases within 2 rows	Attack rate in 2 rows (%)	Wearing mask	Evidence level
Chen [2]	Jan 24, 2020	4 h 50 m	335	332	3	13	3.9	8	14.3	Yes^a^	Low
Khanh [12]	Mar 1, 2020	10 h	201	167	1	14	7	12	80	No^b^	Medium
Hoehl [13]	Mar 9, 2020	4 h 40 m	102	95	7	2	2.6	2	8.3	NA	Low
Speake [14]	Mar 19, 2020	5 h	241	64	18	11	4.9	8	5	No^c^	High
Swadi [15]	Sep 29, 2020	18 h 2 m	86	84	2	4	4.8	4	30.8	Partly^d^	High
Lv [16]	Jun 9, 2021	NA	203	197	6	29	14.7	19	16.4	Yes^e^	High
Toyokawa [17]	Mar 23, 2020	2 h	142	122	1	14	10	7	30.4	Yes^f^	High
Ngeh [18]	Jul 1, 2020	10 h	95	93	2	15	16.1	7	28.0	Mostly^g^	High
Total				1,154	40	102	7.6	67	15.5		

NA, not available; months are abbreviated using the first three letters.

a Passengers removed masks when they ate food or drank water.

b Masks were not widely used on airplanes in early March, particularly among the European travellers.

c Mask use was rare.

d Mask use was not peremptory; Passengers D and F reported mask use but C and E did not.

e 87.3% (172/197) of passengers had taken off their masks during flight.

f Most passengers used masks all times, while the index cases did not.

g Among the 71 passengers interviewed, most of them (68) wore masks, and only three did not.

### Flight attack rate and infection risk

**Figure 2.**
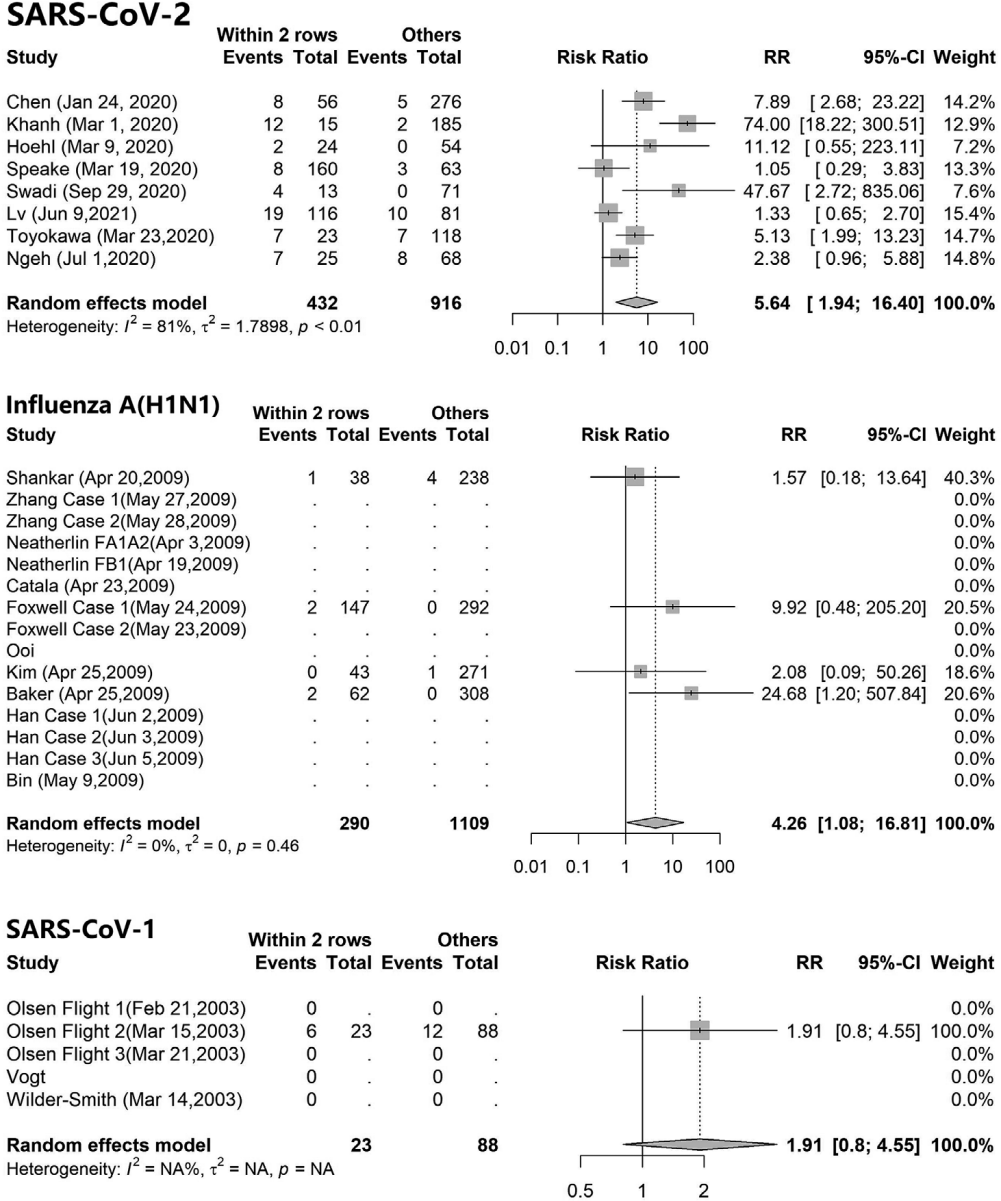
The infection risk ratio of passengers within and beyond two rows of the index case(s) in flights which carried. (a) SARS-CoV-2 cases; (b) influenza A(H1N1) pdm09 virus cases; (c) SARS-CoV-1 cases.

As shown in Table 1, a total of 40 index cases infected 102 of the 1,348 passengers, with an overall attack rate of 7.6%. In the eight flights, the inflight SARS-CoV-2 attack rates ranged from 2.6% to 16.1%. Among the 102 secondary cases of COVID-19, 67 (66%) cases were within two rows of the index cases. The risk of SARS-CoV-2 infection among passengers in the exposure group was 5.6 times that of passengers in the control group (risk ratio 5.64, 95% confidence interval (CI):1.94–16.40), as shown in Figure 2a. The heterogeneity test results showed that *I* ^2^ was 81%, suggesting significant heterogeneity between studies. The funnel plot (Supplementary Figure S1) showed that the heterogeneity was mainly derived from the three studies of Khanh et al. [12], Speake et al. [14], and Lv et al. [16]. The results of the sensitivity analysis (Supplementary Figure S2) indicated that the risk ratios remained significant after the deletion of any study. One inflight COVID-19 outbreak reported by Lv et al. [16] occurred in 2021, when some passengers might have been vaccinated. After deleting this study, passengers in the exposure group had an increased infection risk compared to passengers in the control group (risk ratio 7.32, 95%CI:2.33–23.03).

In 15 influenza A(H1N1)pdm09 virus inflight outbreaks, the attack rates were 0%–5.8% (Supplementary Table S1). The infection risk of the influenza A(H1N1)pdm09 virus among passengers in the exposure group was 4.2 times that of passengers in the control group (risk ratio 4.26, 95%CI:1.08–16.81), as shown in Figure 2b.

In the four flights with SARS-CoV-1 outbreaks, only one had secondary cases. The attack rate for this flight was 16.2%. The risk ratio of SARS-CoV-1 infection was 1.91 (95%CI:0.80–4.55), but this was not statistically significant (Figure 2c).

### Face mask against SARS-CoV-2 transmission

In the eight reported inflight outbreaks, due to a lack of data on mask-wearing in some studies or unclear data in this part, we used those studies with relatively complete information on mask-wearing for comparison. We found that in the two flights with mask use (Chen et al. [2], Lv et al. [16]), the mean SARS-CoV-2 attack rate was 7.94% (42/529). In the flights without mask use (Khanh et al. [12], Speake et al. [14]), the mean attack rate was 5.91% (25/423), which was not significantly different from the flights with mask use (*p* = 0.22).

## Discussion

To the best of our knowledge, this is the first literature review that focused on exploring the transmission routes of SARS-CoV-2. Although there have been some literature reviews of inflight SARS-CoV-2 transmission, these have mainly studied the attack rate of SARS-CoV-2 or the protective effect of masks [19]. This is also the first comparative analysis of the inflight transmission of SARS-CoV-2, SARS-CoV-1, and influenza A(H1N1) pdm09 virus to explore the potential transmission routes of SARS-CoV-2.

Based on the results of the meta-analysis, we found that the inflight transmission feature of SARS-CoV-2 was similar to the influenza A(H1N1)pdm09 virus, rather than SARS-CoV-1. In both SARS-CoV-2 and influenza A(H1N1)pdm09 virus inflight outbreaks, there was significant case clustering proximity to index cases, while in the SARS-CoV-1 outbreak, this was not observed. It showed that both SARS-CoV-2 and influenza A(H1N1)pdm09 virus infections in the airplane cabins were mainly proximity infections, which occurred within 2 meters of the source of infection. Proximity infection has always been considered evidence of droplet transmission routes. Liu et al. [20] found that when a susceptible person is within 1.5 meters of the source of infection, the exposure to droplet nuclei is greatly increased, which is called the proximity effect. However, a modeling study suggested that the short-range airborne route (airborne transmission that occurs close to the source of infection) is mainly the exposure mode of respiratory infection during close contact [7]. Thus, both droplet routes and short-range airborne routes can result in proximity infection.

In the droplet route, infected passengers mainly excrete large droplets containing viruses. However, owing to size and weight limitations, large droplets may fall on surfaces within 2 meters of the infected person [7] or be blocked by seats and other structures. Therefore, the transmission of droplets is mainly limited to within 2 meters of the index case, and passengers outside of two rows of the index case are less likely to be infected in the aircraft cabins. Thus, the infection risk of passengers inside and outside the two rows is very high. For the short-range airborne route, passengers who were infected can produce aerosols containing viral particles [21], and susceptible individuals will be infected after inhaling these aerosols. Therefore, all passengers in the same airplane cabin might be at risk of infection. Owing to the influence of aerosol deposition, air movement, ventilation, and other factors, the closer to the infected person, the greater the risk of infection. However, owing to the special air circulation mode in the aircraft cabin, the supply air is released through air supply ports located on the sides and top of the cabin, leading to the formation of two circulating vortices above the seats. Subsequently, the recirculated air is discharged through the air outlets positioned beneath the cabin seats. Consequently, the airflow is mainly restricted to circulate within the same row of seats [22]. Moreover, the air change rate in air cabins can range from 15 to 20 per hour due to small volume [23], and the high-efficiency particulate air filter used in the airplanes can remove 99.97% of the particles with a radius larger than 0.3 μm [24], so the exhaled droplets can be removed fairly easily. Long-range airborne transmission (airborne transmission that occurs at a greater distance from the source of infection), which is susceptible to ventilation, is less likely to occur in the aircraft cabin than short-range airborne transmission. Passengers within the two rows were more at risk of infection than those outside the two rows. Therefore, both droplet routes and short-range airborne routes might be the main transmission routes of SARS-CoV-2 and influenza A(H1N1)pdm09 virus inflight infection.

**Figure 3.**
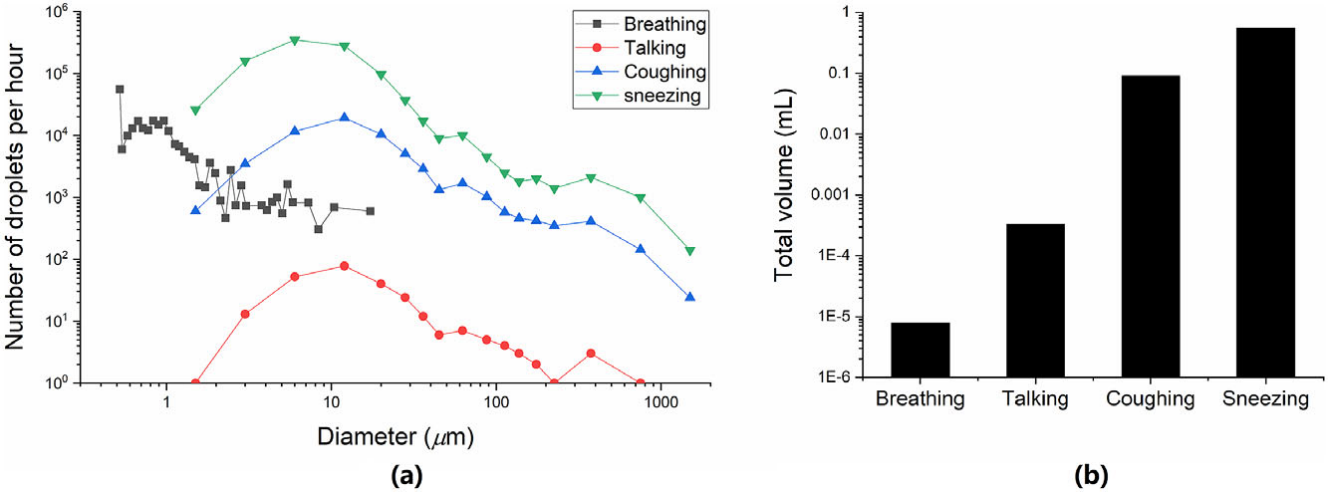
Droplets exhaled from breathing, talking (counting 1–100 once), and coughing per hour. (a) Droplet concentration at different sizes; (b) the total volume, by assuming that, cough frequency was 12/h, and the talking time was about 100 seconds.

Since particle size plays a key role in the relative importance of different routes of respiratory infection transmission, we calculated the distribution of the number and volume of exhaled droplets during different respiratory activities based on the studies of Morawska et al. [25] and Chen et al. [7]. The results are shown in Figure 3. Compared with the particles exhaled from sneezing, coughing, and talking, the volume of particles exhaled from breathing is much smaller. Thus, if virus-containing droplets are mainly exhaled from breathing, the airborne route may play an important role. On the other hand, if virus-containing droplets are mainly exhaled from coughing or sneezing, droplet or fomite routes may become important.

Studies have shown that both influenza A(H1N1)pdm09 virus and SARS-CoV-2 are mainly transmitted by asymptomatic/pre-symptomatic infections. 45%–75% of the influenza A(H1N1)pdm09 virus infections are transmitted by asymptomatic/pre-symptomatic cases [26]. The mean proportion of asymptomatic/pre-symptomatic SARS-CoV-2 was 57.5% [27]. In addition, the peak viral shedding of the two viruses happened before or during the onset of symptoms [28]. This implies that these patients may shed many viral particles via ordinary respiratory activities such as speaking or breathing. Normal breathing only produces aerosols of less than 2 μm [21], and speaking mainly produces droplets of 1–5 μm [25], which causes the virus to spread mainly through the air. Therefore, the short-range airborne route is more appropriate to explain the inflight outbreaks of SARS-CoV-2 and influenza A(H1N1)pdm09 virus. In contrast, asymptomatic/pre-symptomatic SARS-CoV-1 infections only contributed 13% [29], and the peak viral shedding occurred several days after symptom onset [30]. This shows that these patients usually shed many viral particles via coughing and sneezing. Therefore, the droplet route is likely to be the main transmission route of SARS-CoV-1 inflight infection. However, since there are fewer studies on SARS-CoV-1 inflight infection and only one flight was included in this study, the main route of SARS-CoV-1 inflight transmission could not be confirmed.

In this study, the protective ability of masks to protect passengers during flight was not significant. Surgical face masks can filter more than 98% of the large droplets over 5 μm in diameter, while it can only filter about 60% of airborne droplets [31]. We found that, in the flights with mask use, the attack rates of SARS-CoV-2 were not significantly different from those without (*p* = 0.22). Of course, the results may be related to several factors, such as insufficient sample size, incorrect methods of mask-wearing, inadequate time of mask usage, and mask types.

Our study has some limitations, mainly related to the lack of key data in the original study and the varying quality of the studies. First, because there is currently no unified reporting standard for studies reporting inflight transmission of SARS-CoV-2, many studies lack some key inflight infection data. For example, information such as flight duration, aircraft type, seat map, and use of masks is missing. Therefore, we could not perform subgroup analysis to further improve the accuracy of the meta-analysis. Simultaneously, there may be publication bias in this study. As the analysis primarily relied on published studies, it is plausible that studies featuring a smaller number of infected passengers or those reporting non-significant risk ratios may not have been published. This could introduce a bias in the overall findings and conclusions drawn from the study. Second, we assumed that the infection of the secondary case occurred during the period when the passenger was sitting in the seat after boarding, but it is possible that the infection occurred outside the seat, such as in the departure lounge, during boarding and disembarking, or when they leave their seats to go to the toilet during the flight. If susceptible individuals are predominantly infected in these areas, the distribution of secondary cases would display a more random pattern rather than clustering around the index case. This can be attributed to the randomised nature of contact among unfamiliar passengers in both the departure and the arrival halls, and passengers typically not strictly adhering to the seating arrangement when boarding, disembarking, or moving in the aircraft during the flight. Additionally, compared with subways and buses, passengers on planes moved around less frequently and stayed longer in their seats. Therefore, the passenger infection was more likely to occur in the seat. Third, the possible improvement of aircraft ventilation systems by airlines after the outbreak of COVID-19 was not considered, which might influence the comparison between different viruses. However, this assumption had less impact on our main conclusions, because ventilation mainly affects long-range airborne transmission rather than short-range airborne transmission. At last, we cannot deny the possibility that passengers may be infected through exposure to fomites of the index case in the cabin environment surface. Therefore, it is also likely that some infections occurring outside the two rows were caused by exposure to fomites produced in common areas by the index case.

## Conclusions

Based on the result of the meta-analysis, our study suggests that SARS-CoV-2 has a similar transmission characteristic to the influenza A(H1N1)pdm09 virus. The transmission of the two viruses on an aircraft mainly occurs at a close range, and short-range airborne transmission may play an important role in it. In the future, the inflight infection of SARS-CoV-2 should be controlled mainly by blocking the short-range airborne and droplet transmission route. In addition, most published studies on SARS-CoV-2 on-board transmission to date are of varying quality. For future research, a unified standard reporting format should be developed to ensure the integrity of information. At the same time, some quantitative research is also needed, such as using CFD-related software to simulate the movement of virus aerosols and quantify the infection risk of susceptible individuals to obtain more definite conclusions.

## Data Availability

The datasets used for this study are available on request from the corresponding author.

## Supplementary material

For supplementary material accompanying this paper visit https://doi.org/10.1017/S0950268823001012. click here to view supplementary material

## References

[1] WHO (2023) *WHO Coronavirus (COVID-19) Dashboard.* Available at https://covid19.who.int/ (accessed 26 April 2023).

[2] Chen J, He H, Cheng W, Liu Y, Sun Z, Chai C, Kong Q, Sun W, Zhang J, Guo S, Shi X, Wang J, Chen E and Chen Z (2020) Potential transmission of SARS-CoV-2 on a flight from Singapore to Hangzhou, China: An epidemiological investigation. Travel Medicine and Infectious Disease 36, 101816. 10.1016/j.tmaid.2020.101816 32645477 PMC7336905

[3] Murphy N, Boland M, Bambury N, Fitzgerald M, Comerford L, Dever N, O’Sullivan MB, Petty-Saphon N, Kiernan R, Jensen M and O’Connor L (2020) A large national outbreak of COVID-19 linked to air travel, Ireland, summer 2020. Eurosurveillance 25, 2001624. 10.2807/1560-7917.ES.2020.25.42.2001624 33094715 PMC7651877

[4] Lei H, Tang JW, Li Y (2018) Transmission routes of influenza A(H1N1)pdm09: Analyses of inflight outbreaks. Epidemiology and Infection 146, 1731–1739. 10.1017/S0950268818001772 29954469 PMC9507947

[5] Duguid JP (1946). The size and the duration of air-carriage of respiratory droplets and droplet-nuclei. Epidemiology & Infection 44, 471–479. 10.1017/S0022172400019288 PMC2234804 20475760

[6] Stadnytskyi V, Bax CE, Bax A and Anfinrud P (2020). The airborne lifetime of small speech droplets and their potential importance in SARS-CoV-2 transmission. Proceedings of the National Academy of Sciences 117, 11875–11877. 10.1073/pnas.2006874117 PMC7275719 32404416

[7] Chen W, Zhang N, Wei J, Yen HL and Li Y(2020) Short-range airborne route dominates exposure of respiratory infection during close contact. Building and Environment 176, 106859. 10.1016/j.buildenv.2020.106859

[8] US Centers for Disease Control and Prevention (2021) *Scientific Brief: SARS-CoV-2 Transmission.* Available at https://www.cdc.gov/coronavirus/2019-ncov/science/science-briefs/sars-cov-2-transmission.html (accessed 2 June 2023). 34009775

[9] Flerlage T, Boyd DF, Meliopoulos V, Thomas PG and Schultz-Cherry S (2021) Influenza virus and SARS-CoV-2: Pathogenesis and host responses in the respiratory tract. Nature Reviews Microbiology 19, 425–441. 10.1038/s41579-021-00542-7 33824495 PMC8023351

[10] Page MJ, McKenzie JE, Bossuyt PM, Boutron I, Hoffmann TC, Mulrow CD, Shamseer L, Tetzlaff JM, Akl EA, Brennan SE, Chou R, Glanville J, Grimshaw JM, Hróbjartsson A, Lalu MM, Li T, Loder EW, Mayo-Wilson E, McDonald S, McGuinness LA, Stewart LA, Thomas J, Tricco AC, Welch VA, Whiting P and Moher D (2021) The PRISMA 2020 statement: An updated guideline for reporting systematic reviews. British Medical Journal 372, n71. 10.1136/bmj.n71 33782057 PMC8005924

[11] Browne A, Ahmad SS-O, Beck CR, Nguyen-Van-Tam JS (2016) The roles of transportation and transportation hubs in the propagation of influenza and coronaviruses: A systematic review. Journal of Travel Medicine 23, tav002. 10.1093/jtm/tav002 26782122 PMC7539332

[12] Khanh NC, Thai PQ, Quach HL, Thi NAH, Dinh PC, Duong TN, Mai LTQ, Nghia ND, Tu TA, Quang LN, Quang TD, Nguyen TT, Vogt F and Anh DD (2020) Transmission of SARS-CoV 2 during long-haul flight. Emerging Infectious Diseases 26, 2617–2624. 10.3201/eid2611.203299 32946369 PMC7588538

[13] Hoehl S, Karaca O, Kohmer N, Westhaus S, Graf J, Goetsch U and Ciesek S (2020) Assessment of SARS-CoV-2 transmission on an international flight and among a tourist group. JAMA Network Open 3, e2018044. 10.1001/jamanetworkopen.2020.18044 PMC7435338 32809029

[14] Speake H, Phillips A, Chong T, Sikazwe C, Levy A, Lang J, Scalley B, Speers DJ, Smith DW, Effler P and McEvoy SP (2020) Flight-associated transmission of severe acute respiratory syndrome coronavirus 2 corroborated by whole-genome sequencing. Emerging Infectious Diseases 26, 2872–2880. 10.3201/eid2612.203910 32990563 PMC7706937

[15] Swadi T, Geoghegan JL, Devine T, McElnay C, Sherwood J, Shoemack P, Ren X, Storey M, Jefferies S, Smit E, Hadfield J, Kenny A, Jelley L, Sporle A, McNeill A, Reynolds GE, Mouldey K, Lowe L, Sonder G, Drummond AJ, Huang S, Welch D, Holmes EC, French N, Simpson CR and de Ligt J (2021) Genomic evidence of in-flight transmission of SARS-CoV-2 despite predeparture testing. Emerging Infectious Diseases 27, 687–693. 10.3201/eid2703.204714 33400642 PMC7920679

[16] Lv Q, Kong D, He Y, Lu Y, Chen L, Zhao J, Feng S, Chen Y, Wan J, Wen Y, Gao W, Chen Z, Tang X, Mei S, Zou X and Feng T (2021) A SARS-CoV-2 delta variant outbreak on airplane: Vaccinated air passengers are more protected than unvaccinated. Journal of Travel Medicine 28, taab161. 10.1093/jtm/taab161 34609488 PMC8522384

[17] Toyokawa T, Shimada T, Hayamizu T, Sekizuka T, Zukeyama Y, Yasuda M, Nakamura Y, Okano S, Kudaka J, Kakita T, Kuroda M and Nakasone T (2022) Transmission of SARS-CoV-2 during a 2-h domestic flight to Okinawa, Japan, March 2020. Influenza and Other Respiratory Viruses 16, 63–71. 10.1111/irv.12913/34605181 PMC8652895

[18] Ngeh S, Vogt F, Sikazwe CT, Levy A, Pingault NM, Smith DW and Effler PV (2022) Travel-associated SARS-CoV-2 transmission documented with whole genome sequencing following a long-haul international flight. Journal of Travel Medicine 29, taac057. 10.1093/jtm/taac057 35532195 PMC9129214

[19] Freedman DO, Wilder-Smith A (2020) In-flight transmission of SARS-CoV-2: A review of the attack rates and available data on the efficacy of face masks. Journal of Travel Medicine 27, taaa178. 10.1093/jtm/taaa178 32975554 PMC7543400

[20] Liu L, Li Y, Nielsen PV, Wei J and Jensen RL (2017) Short-range airborne transmission of expiratory droplets between two people. Indoor Air 27, 452–462. 10.1111/ina.12314 27287598

[21] Scheuch G (2020) Breathing is enough: For the spread of influenza virus and SARS-CoV-2 by breathing only. Journal of Aerosol Medicine and Pulmonary Drug Delivery 33, 230–234. 10.1089/jamp.2020.1616 32552296 PMC7406993

[22] Wang W, Wang F, Lai D and Chen Q (2022) Evaluation of SARS-CoV-2 transmission and infection in airliner cabins. Indoor Air 32, e12979. 10.1111/ina.12979 35048429

[23] Mangili A, Gendreau MA (2005) Transmission of infectious diseases during commercial air travel. Lancet 365, 989–996. 10.1016/S0140-6736(05)71089-8 15767002 PMC7134995

[24] Lindgren T, Norbäck D (2002) Cabin air quality: Indoor pollutants and climate during intercontinental flights with and without tobacco smoking. Indoor Air 12, 263–272. 10.1034/j.1600-0668.2002.01121.x 12532758

[25] Morawska L, Johnson GR, Ristovski ZD, Hargreaves M, Mengersen K, Corbett S, Chao CYH, Li Y and Katoshevski D (2009) Size distribution and sites of origin of droplets expelled from the human respiratory tract during expiratory activities. Journal of Aerosol Science 40, 256–269. 10.1016/j.jaerosci.2008.11.002 PMC7126899 32287373

[26] Hayward AC, Fragaszy EB, Bermingham A, Wang L, Copas A, Edmunds WJ, Ferguson N, Goonetilleke N, Harvey G, Kovar J, Lim MS, McMichael A, Millett ER, Nguyen-van-Tam J, Nazareth I, Pebody R, Tabassum F, Watson JM, Wurie FB, Johnson AM, Zambon M and **Flu Watch Group** (2014) Comparative community burden and severity of seasonal and pandemic influenza: Results of the Flu Watch cohort study. Lancet Respiratory Medicine 2, 445–454. 10.1016/S2213-2600(14)70034-7 24717637 PMC7164821

[27] Yanes-Lane M, Winters N, Fregonese F, Bastos M, Perlman-Arrow S, Campbell JR and Menzies D (2020) Proportion of asymptomatic infection among COVID-19 positive persons and their transmission potential: A systematic review and meta-analysis. PLoS One 15, e0241536. 10.1371/journal.pone.0241536 33141862 PMC7608887

[28] Wu Z, Harrich D, Li Z, Hu D and Li D (2021) The unique features of SARS-CoV-2 transmission: Comparison with SARS-CoV, MERS-CoV and 2009 H1N1 pandemic influenza virus. Reviews in Medical Virology 31, e2171. 10.1002/rmv.2171 33350025 PMC7537046

[29] Wilder-Smith A, Teleman MD, Heng BH, Earnest A, Ling AE and Leo YS (2005) Asymptomatic SARS coronavirus infection among healthcare workers, Singapore. Emerging Infectious Diseases 11, 1142–1145. 10.3201/eid1107.041165 16022801 PMC3371799

[30] Peiris JS, Chu CM, Cheng VC, Chan KS, Hung IF, Poon LL, Law KI, Tang BS, Hon TY, Chan CS, Chan KH, Ng JS, Zheng BJ, Ng WL, Lai RW, Guan Y, Yuen KY and **HKU/UCH SARS Study Group** (2003) Clinical progression and viral load in a community outbreak of coronavirus-associated SARS pneumonia: A prospective study. Lancet 361, 1767–1772. 10.1016/s0140-6736(03)13412-5 12781535 PMC7112410

[31] Koh XQ, Sng A, Chee JY, Sadovoy A, Luo P and Daniel D (2022) Outward and inward protection efficiencies of different mask designs for different respiratory activities. Journal of Aerosol Science 160, 105905. 10.1016/j.jaerosci.2021.105905

