# Supplementary material for: Comparative analysis of inflight transmission of SARS-CoV-2, influenza, and SARS-CoV-1
Source: Epidemiol Infect. 2023 Jun 23;151:e111. doi: 10.1017/S0950268823001012 (PMC10344696; doi:10.1017/S0950268823001012)
Supplement: Supplementary file 1 [file S0950268823001012sup001.docx]

**Supplementary Material**

**Search strategies**:

We searched the PubMed databases for air travel-associated transmission of SARS-CoV-2 infection January 1, 2020, to July 2, 2022, using the following strategies:

#1 ((((((((((((((((((((((((aircraft[Title/Abstract]) OR aeroplane[Title/Abstract]) OR airline[Title/Abstract]) OR flight[Title/Abstract]) OR aircrew[Title/Abstract]) OR airflight[Title/Abstract]) OR airplane[Title/Abstract]) OR aviation[Title/Abstract]) OR airport[Title/Abstract]) OR aeroport[Title/Abstract]) OR aero transport[Title/Abstract]) OR airport[Title/Abstract]) OR steward[Title/Abstract]) OR stewardess[Title/Abstract]) OR flight[Title/Abstract]) OR flights[Title/Abstract]) OR inflight[Title/Abstract]) OR in-flight[Title/Abstract]) OR cabin crew[Title/Abstract]) OR cabin[Title/Abstract]) OR cabins[Title/Abstract]) OR fly[Title/Abstract]) OR flying[Title/Abstract]) OR tour[Title/Abstract]) OR travel[Title/Abstract]) OR journal[Title/Abstract]) OR passenger[Title/Abstract])

#2 ((((((COVID-19[Title/Abstract]) OR SARS-CoV-2[Title/Abstract]) OR coronavirus 2019[Title/Abstract]) OR coronavirus disease 2019[Title/Abstract]))

#3 ("2020/01/01"[Date - Publication]: "3000"[Date - Publication])

#4 #1 AND #2 AND #3

Limits: not limits

**Figure S1.** Funnel plot results of the of the SARS-CoV-2 flight transmission meta-analysis.

**
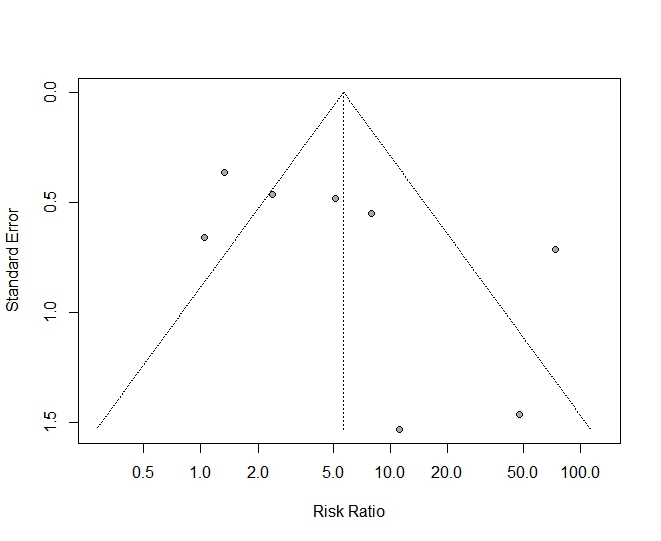
**

**Figure S2.** Sensitivity analysis results of the SARS-CoV-2 flight transmission meta-analysis.


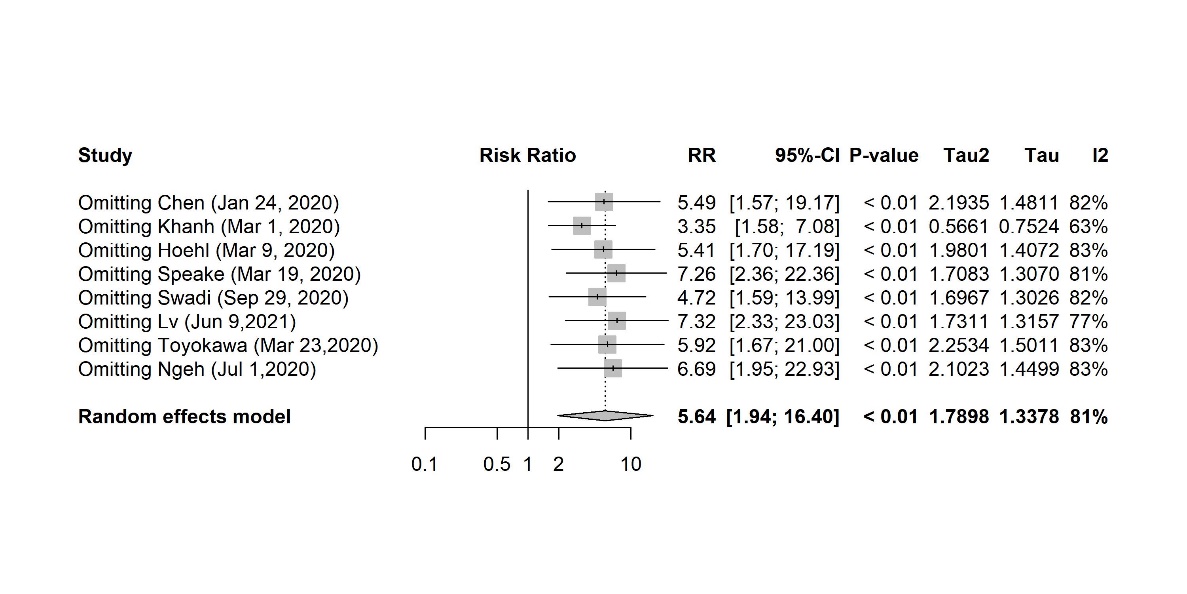


**Supplementary Table S1.** Summary of inflight influenza A(H1N1) pdm09 virus transmission from literatures

| **First author** | **Departure date** | **Flight duration** | **Passengers Aboard** | **Passengers Traced** | **Index Cases** | **Secondary Cases Identified** | **Attack Rate %** | **Secondary Cases Within 2 Rows** | **Attack Rate in 2 Rows %** | **Diagnosis method**^#^ | **Include in meta analysis** |
| --- | --- | --- | --- | --- | --- | --- | --- | --- | --- | --- | --- |
| Shankar ^[1]^ | Apr 20, 2009 | 9.5 h | 278 | 43 | 1 | 5 | 1.8 | 1 | 2.6 | L | Yes |
| Zhang ^[2]^  Case 1 | May 27, 2009 | 20h 20m | 260 | 82 | 1 | 9 | 3.5 | NA | NA | L | No |
| Zhang ^[2]^  Case 2 | May 28, 2009 | 1h 40m | 136 | 136 | 1 | 0 | 0 | 0 | 0 | L | No |
| Neatherlin^[3]^Case 1 | Apr 3, 2009 | 3h 20m | 265 | 146 | 1 | 8 | 3.0 | 3 | 6.5 | L | No |
| Neatherlin^[3]^ Case 2 | Apr 19, 2009 | 4h 5m | 129 | 121 | 1 | 6 | 5.8 | 3 | 14.3 | S | No |
| Catala^[4]^ | Apr 23, 2009 | 11h | 165 | 159 | 6 | 4 | 2.5 | 2 | NA | L | No |
| Foxwell^[5]^ Case 1 | Mar 24, 2009 | 14h | 445 | 188 | 6 | 2 | 0.5 | 2 | 1.4 | L | Yes |
| Foxwell^[5]^ Case 2 | Mar 23, 2009 | 8h | 293 | 131 | NA | 1 | NA | 4 | NA | S+L | No |
| Ooi^[6]^ | NA | NA | 596 | 25 | 1 | 5 | 0.8 | 2 | NA | L | No |
| Kim ^[7]^ | Apr 25, 2009 | 13h | 315 | 176 | 1 | 1 | 0.3 | 0 | 0 | L | Yes |
| Baker ^[8]^ | Apr 25, 2009 | 13h | 379 | 121 | 9 | 2 | 0.5 | 2 | 3.2 | L | Yes |
| Han ^[9]^ Case 1 | Jun 2, 2009 | 2h 35m | 92 | 91 | 1 | 0 | 0 | 0 | 0 | L | No |
| Han ^[9]^ Case 2 | Jun 3, 2009 | 45m | 111 | 87 | 1 | 0 | 0 | 0 | 0 | L | No |
| Han ^[9]^ Case 3 | Jun 5, 2009 | 45m | 111 | 87 | 2 | 1 | 1.2 | 1 | NA | L | No |
| Bin ^[10]^ | May 9, 2009 | NA | 142 | 141 | 1 | 0 | 0 | 0 | 0 | L | No |
| Total^*^ |  |  | 1417 | 528 | 17 | 10 | 0.7 | 5 | 1.7 |  |  |

NA, not available, months are abbreviated using the first three letters.

* Only studies included in the meta-analysis are included.

^#^L=laboratory confirmed, S=symptoms confirmed.

**Supplementary Table S2.** Summary of inflight SARS-CoV-1 transmission from literatures.

| **First author** | **Departure date** | **Flight duration** | **Passengers Aboard** | **Passengers**  **Traced** | **Index Cases** | **Secondary Cases Identified** | **Attack Rate %** | **Secondary Cases Within 2 Rows** | **Attack Rate in 2 Rows %** | **Include in meta analysis** |
| --- | --- | --- | --- | --- | --- | --- | --- | --- | --- | --- |
| Olsen ^[11]^ Flight 1 | Feb 21, 2003 | 1h 30m | 315 (including crew) | 74 | 1 | 0 | 0 | 0 | 0 | No |
| Olsen ^[11]^  Flight 2 | Mar 15, 2003 | 3h | 112 | 65 | 1 | 18 | 16.2 | 6 | 26.1 | Yes |
| Olsen ^[11]^ Flight 3 | Mar 21, 2003 | 1h 30m | 246(including crew) | 166 | 4 | 0 | 0 | 0 | 0 | No |
| Vogt ^[12]^ | 7 flights with 1 index patient per flight | NA | 1766 contacted | 312 interviewed/ 127 serology | 1 per flight | 0 | 0 | 0 | 0 | No |
| Wilder-  Smith ^[13]^ | Mar 14, 2003 | NA | NA | NA | 1 | 0 | 0 | 0 | 0 | No |
| Total^*^ |  |  | 112 | 65 | 1 | 18 | 16.2 | 6 | 26.1 |  |

NA, not available, months are abbreviated using the first three letters.

^*^ Only studies included in the meta-analysis are included.

**References**

[1] **Shankar AG, et al.** (2014) Contact tracing for influenza A(H1N1) pdm09 virus-infected passenger on international flight. *Emerging infectious diseases*; **20**:118-120. doi:10.3201/eid2001.120101.

[2] **Zhang L, et al.** (2013) Protection by face masks against influenza A(H1N1) pdm09 virus on trans-Pacific passenger aircraft, 2009. *Emerging infectious diseases*; **19**:1403. doi:10.3201/eid1909.121765.

[3] **Neatherlin J, et al.** (2013) Influenza A(H1N1) pdm09 during air travel. *Travel Medicine and Infectious Disease*; **11**:110-118. doi:10.1016/j.tmaid.2013.02.004.

[4] **Catala L, et al.** (2012) Pandemic A/H1N1 influenza: transmission of the first cases in Spain. *Enfermedades Infecciosas y Microbiología Clínica*; **30**:60-63. doi:10.1016/j.eimc.2011.06.007.

[5] **Foxwell AR, et al.** (2011) Transmission of influenza on international flights, may 2009. *Emerging infectious diseases*; **17**:1188-1194. doi:10.3201/eid1707.101135.

[6] **Ooi PL LF, et al.** (2010) Clinical and molecular evidence for transmission of novel influenza A(H1N1/2009) on a commercial airplane. *Archives of Internal Medicine*; **170**:913-915. doi:10.1001/archinternmed.2010.127.

[7] **Kim JH, et al.** (2010) In-Flight Transmission of Novel Influenza A (H1N1). *Epidemiology and Health*; **32**: e2010006. doi:10.4178/epih/e2010006.

[8] **Baker MG, et al.** (2010) Transmission of pandemic A/H1N1 2009 influenza on passenger aircraft: retrospective cohort study. *British Medical Journal*; **340**:c2424. doi:10.1136/bmj.c2424.

[9] **Han K, et al.** (2009) Lack of airborne transmission during outbreak of pandemic (H1N1) 2009 among tour group members, China, June 2009. *Emerging infectious diseases*; **15**: 1578-1581. doi:10.3201/eid1510.091013.

[10] **Bin C, et al.** (2009) Clinical and epidemiologic characteristics of 3 early cases of influenza A pandemic (H1N1) 2009 virus infection, People's Republic of China, 2009. *Emerging infectious diseases*; **15**:1418-1422. doi:10.3201/eid1509.090794.

[11] **Olsen SJ, et al.** (2003) Transmission of the severe acute respiratory syndrome on aircraft. *New England Journal of Medicine*; **349**:2416-2422. doi:10.1056/NEJMoa031349.

[12] **Vogt TM, et al.** (2006) Risk of severe acute respiratory syndrome-associated coronavirus transmission aboard commercial aircraft. *Journal of Travel Medicine*; **13**:268-272. doi:10.1111/j.1708-8305.2006.00048.x.

[13] **Wilder-Smith A LH, Villacian JS.** (2003) In-flight transmission of Severe Acute Respiratory Syndrome(SARS): a case report. *Journal of Travel Medicine*; **10**:299-300. doi:10.2310/7060.2003.2737.
